# Effect of auditory-motor training on auditory processing of school children

**DOI:** 10.31744/einstein_journal/2018AO4359

**Published:** 2018-11-29

**Authors:** Fátima Aparecida Gonçalves, Márcia Ribeiro Vieira, Liliane Desgualdo Pereira

**Affiliations:** 1 Departamento de Fonoaudiologia, Escola Paulista de Medicina, Universidade Federal de São Paulo, São Paulo, SP, Brazil.

**Keywords:** Auditory perception, Hearing disorders, Neuronal plasticity, Physical stimulation, Exercise therapy, Child, Percepção auditiva, Transtornos da audição, Plasticidade neuronal, Estimulação física, Terapia por exercício, Criança

## Abstract

**Objective:**

To compare performance in *Avaliação Simplificada do Processamento Auditivo Central* and Scale of Auditory Behaviors scores before and after auditory and motor training.

**Methods:**

Sample comprising 162 children aged 9 to 11 years and attending public schools in the city of São Paulo (SP), Brazil; 122 out of 162 children were allocated to one of three experimental groups: Multisensory; Auditory/Motor and Motor/Auditory. Experimental groups were submitted to 8 hours of auditory, visuospatial and motor stimulation over the course of 8 weeks. The remaining 40 children formed the Control Group and received no stimulation.

**Results:**

Relation between child behavior as perceived by school teachers and auditory test responses revealed that the better the performance in auditory processing assessment, the higher the Scale of Auditory Behaviors scores.

**Conclusion:**

Auditory and motor training led to improvements in auditory processing skills as rated by *Avaliação Simplificada do Processamento Auditivo Central* and Scale of Auditory Behaviors; this intervention model proved to be a good tool for use in school settings.

## RESUMO

**Objetivo:**

Comparar as respostas da Avaliação Simplificada do Processamento Auditivo Central às da *Scale of Auditory Behaviors* antes e depois de um treinamento auditivo e de habilidades motoras.

**Métodos:**

Participaram 162 escolares de 9 a 11 anos de escola pública municipal da cidade de São Paulo (SP), sendo 122 deles divididos em três grupos experimentais: Multissensorial; Auditivo/Motor e Motor/Auditivo. Eles receberam estimulação auditiva, visuoespacial e motora durante 8 semanas (8 horas). O Grupo Controle, que não recebeu estimulação, foi formado por 40 escolares.

**Resultados:**

A relação entre a percepção dos professores sobre o comportamento dos alunos e suas respostas em testes auditivos mostrou que quanto melhor o resultado na avaliação do processamento auditivo, melhores os escores da *Scale of Auditory Behaviors*.

**Conclusão:**

Após treinamento auditivo e motor, ocorreu melhora significativa das habilidades do processamento auditivo demonstradas pela Avaliação Simplificada do Processamento Auditivo Central e na *Scale of Auditory Behaviors*, e este modelo de intervenção consistiu em boa ferramenta para uso na escola.

## INTRODUCTION

Around 50% of children attending public primary schools begin high school with significant reading and writing problems, if not illiterate, and 30% present with some kind of learning disorder or difficulty.^(^ ^1^ ^)^

These indices often reflect learning difficulties, writing and logical mathematical reasoning *deficits* in particular. Learning difficulties have been the focus of recent research, given the significance of learning to full human sociocultural and emotional development.^(^ ^2^ ^,^ ^3^ ^)^

Auditory processing is the ability of central auditory pathways to use auditory input effectively, and includes several auditory mechanisms associated with different skills, such as sound localization, temporal resolution and sound sequencing.^(^ ^4^ ^)^ Temporal resolution is critical for speech intelligibility and a requirement for linguistic and reading skills.^(^ ^5^ ^)^ Likewise, sound localization impacts mobility and communication, with significant contributions to selective attention – a vital skill for acquisition of new learning content.^(^ ^6^ ^)^

Studies investigating auditory changes associated with learning disorders^(^ ^3^ ^,^ ^7^ ^-^ ^10^ ^)^ revealed that most affected children have compromised auditory processing.^(^ ^3^ ^,^ ^10^ ^,^ ^11^ ^)^ Reduced ability to use auditory stimuli in speech perception may contribute to reading and writing *deficits*. Therefore, auditory processing assessment plays a key role in identification of school children with learning difficulties and in further interventions.^(^ ^12^ ^-^ ^14^ ^)^

Major complaints in children with central auditory processing disorder (CAPD) include inability to follow complex verbal instructions; poor verbal compared to nonverbal cognitive performance; reading and writing difficulties; speech delay; impaired processing of verbal input in noisy environments, and selected auditory attention deficits.^(^ ^15^ ^)^

Functional central nervous system plasticity, the existence of critical periods for learning, and strengthening of synaptic connections via repetition have been demonstrated in cognitive neuroscience research.^(^ ^16^ ^)^ Also, auditory stimulation and auditory-verbal training are thought to promote functional and structural changes in the central auditory nervous system.^(^ ^7^ ^,^ ^16^ ^-^ ^18^ ^)^ This concept has led to the implementation of CAPD-specific interventions aimed at school children, with particular emphasis on speech therapy, including environmental changes, the therapeutic process *per se* and compensatory strategies.^(^ ^19^ ^,^ ^20^ ^)^

Behavioral tests play a significant role in screening for central auditory processing changes and identification of school children with hearing loss, and evidence of learning disabilities in school settings.^(^ ^7^ ^,^ ^12^ ^,^ ^13^ ^,^ ^21^ ^)^

The *Avaliação Simplificada do Processamento Auditivo Central* (ASPAC) [Simplified Central Auditory Processing Assessment] is a user-friendly screening tool for hearing impairment in school children, which comprises sound localization and verbal and nonverbal sequence memory tests with three to four sounds. Poor performance in ASPAC may indicate auditory perceptual impairment. Early detection of such impairments (*i.e*., at school) may contribute to referral of affected children for comprehensive auditory processing assessment and proper therapeutic intervention.^(^ ^7^ ^,^ ^14^ ^,^ ^21^ ^)^

Since 2010, the American Academy of Audiology (AAA)^(^ ^22^ ^)^ recommends the use of self-perception questionnaires for auditory complaint investigation and qualitative analysis, and to complement behavioral tests in CAPD diagnosis. The Scale of Auditory Behaviors (SAB) in one such questionnaires^(^ ^23^ ^)^ available in Portuguese,^(^ ^24^ ^)^ and allows the quantification of child behaviors associated with auditory processing in everyday life according to parent/teacher perception. Questionnaire items interrogate behaviors such as understanding of verbal instructions, attention quality, speech-sound discrimination, self-organization skills in everyday life and reading, to screen for detectable signs of dysfunction.

A Portuguese study investigated the correlation between SAB scores and performance in behavioral hearing tests, and suggested this tool can be used to screen school children with auditory processing-related learning difficulties.^(^ ^13^ ^)^

This study proposes a therapeutic and educational approach based on auditory and motor stimulation of groups of school children, in school settings, to improve auditory perceptual ability. This study shall contribute to health/education partnerships via therapeutic, educational, multisensory intervention programs aimed at children with learning complaints and no immediate access to specialized therapy.

## OBJECTIVE

To compare performance in the *Avaliação Simplificada do Processamento Auditi*vo *Central* and in the Scale of Auditory Behaviors before and after auditory and motor skill training.

## METHODS

Experimental longitudinal study involving informal training of children attending a public school in the city of São Paulo (SP). This study was approved by the Research Ethics Committee of the *Universidade Federal de São Paulo*, committee opinion no. 542.418, CAAE: 25398314.7.0000.5505.

The sample comprised 162 children (86 females) aged 9 to 11 years, who met all inclusion criteria (age group, proper cognitive function, lack of neurologic and/or psychiatric disorders and informed consent for participation).

All participants completed initial (IA, pre-training) and final (T2, post-training) assessment procedures. The training program in this trial was named Multisensory Stimulation.

### Assessment procedures

#### Avaliação Simplificada do Processamento Auditivo Central (ASPAC)

This simplified central auditory processing assessment comprises exclusively auditory, sound localization and verbal and nonverbal memory sequence tasks. The test for sound localization in five directions consisted of diotic tasks involving high frequency sounds presented in five directions (front, back, above, right and left); participants were asked to point in the direction of sound. This test was used to assess sound localization skills and the physiologic mechanism underlying sound source direction discrimination. Normal auditory processing was defined as four or more correct hits, provided these included the lateral plane.

The Memory Test For Verbal Sequence (MSV) consisted of verbal stimuli (four syllables; “pa”, “ta”, “ca” and “fa”) presented in different sequences. Participants were first asked to repeat each syllable alone, then in the sequence they were spoken. Four instruments (rattle, *coco*, agogo and bell) played in different sequences were used in the memory test for nonverbal sequence. Following a demonstration, participants were asked to close their eyes and point to instruments as they were played. Participants achieving two or more correct hits out of three attempts were defined as normal. Participants failing in this test were exposed to three-sound sequences and the same assessment criterion applied.

#### Scale of Auditory Behaviors

The SAB^(^ ^13^ ^,^ ^23^ ^,^ ^24^ ^)^ consists of a 12-item questionnaire intended for parents and/or school teachers for data collection and CAPD diagnostic support purposes. School teachers in this sample provided answers to questionnaire items (Portuguese version) related to everyday life events reflecting child behavior in the auditory and attentional domains. Behaviors were scored 1.0, 2.0, 3.0, 4.0 and 5.0 (behaviors occurring frequently, most of the time, sometimes, sporadically and never, respectively). Item scores were added up to form a final score ranging from 12 to 60 (total score). In this analysis, SAB scores of 46, 45 to 36 and ≤35 corresponded to normal, suggestive of CAPD and evidence of CAPD, respectively.^(^ ^13^ ^)^

The Multisensory Stimulation training program was designed to provide auditory, visuospatial and motor stimulation to groups of children in school settings. The program comprised 16 biweekly 30-minute sessions (total stimulation time, 8 hours), divided according to stimulation type (single or combined) and applied to three randomly selected groups per classroom. Children were reassessed at the end of each eight-session phase and the type of intervention changed in groups submitted to single stimulation. Groups were named as follows: Auditory/Motor Group (AMG) - 40 children submitted to single stimulation (eight auditory stimulation sessions followed by eight visuospatial and motor stimulation sessions); Motor/Auditory Group (MAG) - 41 children submitted to single stimulation in the opposite order (visuospatial and motor stimulation followed by auditory stimulation); Multisensory Group (MSG) - 41 children submitted to combined auditory, visuospatial and motor stimulation (16 sessions) and Control Group (CG) - 40 non-stimulated children who served as reference for typical development in this environment and age group, and comparison with experimental groups.

The informal training program described was based on Pereira et al.,^(^ ^25^ ^)^ Stimulation procedures employed in each group are described in appendix 1.

Data were expressed as descriptive statistics, with a 0.05 level of significance. Scale of Auditory Behaviors scores consist of small numerical values; however, such values represent a conversion of ordinal variables and allow the distinction between elements in the sample based on element qualities/differences. Therefore, pre- and post-training (IA and T2) intergroup differences were investigated using the non-parametric Wilcoxon test. Correlations between instruments (*i.e*., positive associations between SAB scores and performance in ASPAC – the higher the SAB score, the higher the number of correct responses in ASPAC tests) were investigated using the non-parametric Spearman correlation test. Parametric tests were used and the T value (lowest summed score) calculated to investigate score differences per ASPAC test, per group and calculated p value, to compare different time points (IA *versus* T2) and groups (MAG *versus* AMG *versus* MSG *versus* CG) according to type of intervention. Analysis of variance (ANOVA) was used for IA *versus* T2 comparisons.

## RESULTS

Descriptive statistics of experimental and Control Group SAB scores (final score per group and per time point) are given in table 1.

**Table 1:** Descriptive statistics for Scale of Auditory Behaviors considering the total score per groups studied and time points

Group	Time	n	Maximum	Minimum	Mean	Median	Standard deviation	Q1	Q3	p value IA *versus* T2
**MAG**	AI	41	60	12	40.75	42	12.89	32	49	<0.001*
	T2	41	60	29	47.14	49	8.70	44	54	
**AMG**	AI	40	60	12	48.65	53	12.56	43	59	0.143
	T2	40	60	31	51.07	53	7.90	45.75	58	
**MSG**	AI	41	60	23	46.61	50	12.17	39	58	0.264
	T2	41	60	23	49.02	50	8.39	44	56	
**CG**	AI	40	60	27	47.90	49.5	10.39	42	59.25	0.479
	T2	40	60	22	48.60	51	10.95	44.25	60	

* Statistically significant. Q1: first quartile; Q3: third quartile; IA: initial assessment; T2: final assessment; MAG: Motor/Auditory Group; AMG: Auditory/Motor Group; MSG: Multisensory Group; CG: Control Group.

Pre-training assessment revealed warning signs of CAPD in the MAG, while remaining groups fell within normal ranges (normal SAB scores). All groups scored higher in post- (T2) compared to IA assessments. However, significant differences (T=61; n=41; p<0.001) were limited to the MAG.

Descriptive statistics of sound localization tests (ASPAC MSV4 and MSnV4 tests) per group per time point are given in table 2.

**Table 2:** Descriptive statistics for *Avaliação Simplificada do Processamento Auditivo Central* per groups studied and time points

Group	Tests	Time	n	Minimum	Maximum	Mean	Median	Standard deviation	Q1	Q3	p value IA *versus* T2
**MAG**	LS	AI	41	4	5	4.87	5	0.331	5	5	0.103
		T2	41	4	5	4.97	5	0.156	5	5	
	MSV4s/3	AI	41	0	3	1.95	2	0.947	1	3	0.000*
		T2	41	2	3	2.63	3	0.488	2	3	
	MSnV4s/3	AI	41	0	3	1.90	2	0.889	2	2	0.001*
		T2	41	1	3	2.43	2	0.550	2	3	
**AMG**	LS	AI	40	3	5	4.77	5	0.480	5	5	0.785
		T2	40	4	5	4.80	5	0.405	5	5	
	MSV4s/3	AI	40	0	3	1.85	2	1.210	1	3	0.000*
		T2	40	2	3	2.60	3	0.496	2	3	
	MSnV4s/3	AI	40	0	3	1.80	2	0.883	1	2	0.000*
		T2	40	1	3	2.52	3	0.640	2	3	
**MSG**	LS	AI	41	4	5	4.87	5	0.331	5	5	0.421
		T2	41	4	5	4.92	5	0.264	5	5	
	MSV4s/3	AI	41	0	3	1.85	2	1.108	1	3	0.001*
		T2	41	0	3	2.36	3	0.799	2	3	
	MSnV4s/3	AI	41	0	3	2.04	2	0.921	1	3	0.030*
		T2	41	1	3	2.41	2	0.591	2	3	
**CG**	LS	AI	40	4	5	4.87	5	0.335	5	5	0.486
		T2	40	4	5	4.92	5	0.267	5	5	
	MSV4s/3	AI	40	0	3	2.25	2	0.809	2	3	0.838
		T2	40	0	3	2.23	3	1.012	2	3	
	MSnV4s/3	AI	40	0	3	2.20	2	0.823	2	3	0.030*
		T2	40	0	3	1.82	2	0.970	1	2.5	

* Statistically significant. Q1: first quartile; Q3: Third quartile; IA: initial assessment; T2: final assessment; MAG: Motor/Auditory Group; SL: sound localization; VSM4s/3: verbal sequence memory test - 4 sounds/3 sequences; NVSM4s/3: nonverbal sequence memory test - 4 sounds/3 sequences; AMG: Auditory/Motor Group; MSG: Multisensory Group; CG: Control Group.

Pre- and post- training sound localization performance did not differ significantly between groups. Experimental groups had higher positive MSV and MSnV variations at T2 compared to the CG.

Intergroup pre- and post-training comparisons are shown in table 3.

**Table 3:** Analysis of variance among groups, considering the initial and final time points

ASPAC per time point	SL	NVSM4s/3	VSM4s/3
AI	0.526	0.197	0.262
T2	0.044*	0.001^†^	0.050

Post-hoc comparison using Tukey HDS. * difference between Motor/Auditory Group and Auditory/Motor Group (T=3.906; p=0.029); ^†^ all groups differ from the Control Group.

ASPAC: *Avaliação Simplificada do Processamento Auditivo Central*; SL: sound localization; NVSM4s/3: nonverbal sequence memory test - 4 sounds/3 sequences; VSM4s/3: verbal sequence memory test - 4 sounds/3 sequences; IA: pre-training; T2: post-training.

**Figure 1:**
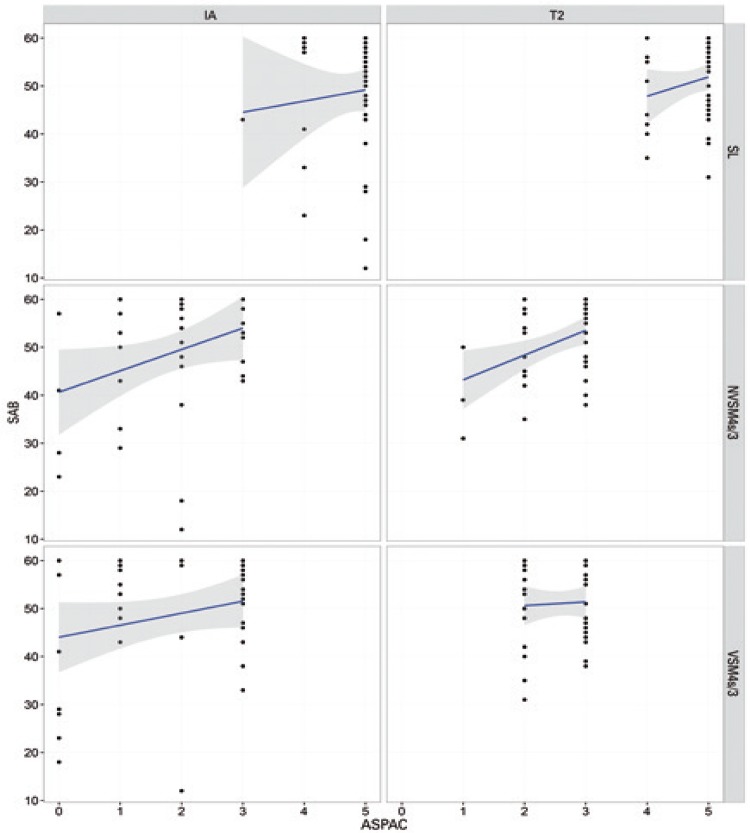
Relation between Scale of Auditory Behaviors scores and performance in *Avaliação Simplificada do Processamento Auditivo Centra*l in the Motor/Auditory Group at initial (IA; left column) and final (T2; right column) time points

SL: sound localization; NVSM4s/3: nonverbal sequence memory test - 4 sounds/3 sequences; VSM4s/3: verbal sequence memory test - 4 sounds/3 sequences; SAB: Scale of Auditory Behaviors; ASPAC: *Avaliação Simplificada do Processamento Auditivo Central*.

**Figure 2:**
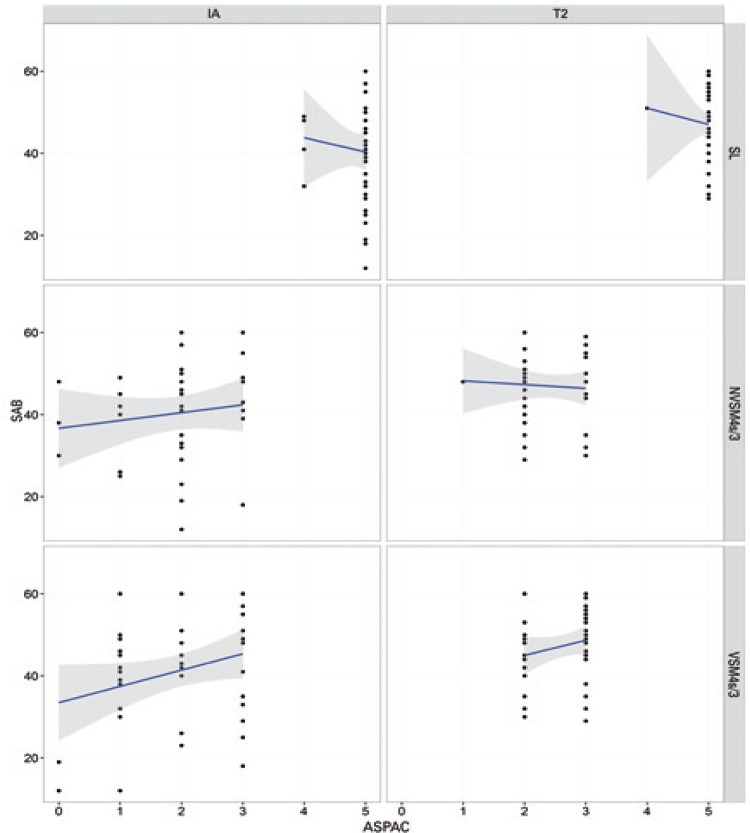
Relation between Scale of Auditory Behaviors scores and performance in *Avaliação Simplificada do Processamento Auditivo Central* in the Auditory/Motor Group at initial (IA; left column) and final (T2; right column) time points

SL: sound localization; NVSM4s/3: nonverbal sequence memory test - 4 sounds/3 sequences; VSM4s/3: verbal sequence memory test - 4 sounds/3 sequences; SAB: Scale of Auditory Behaviors; ASPAC: *Avaliação Simplificada do Processamento Auditivo Central*.

**Figure 3:**
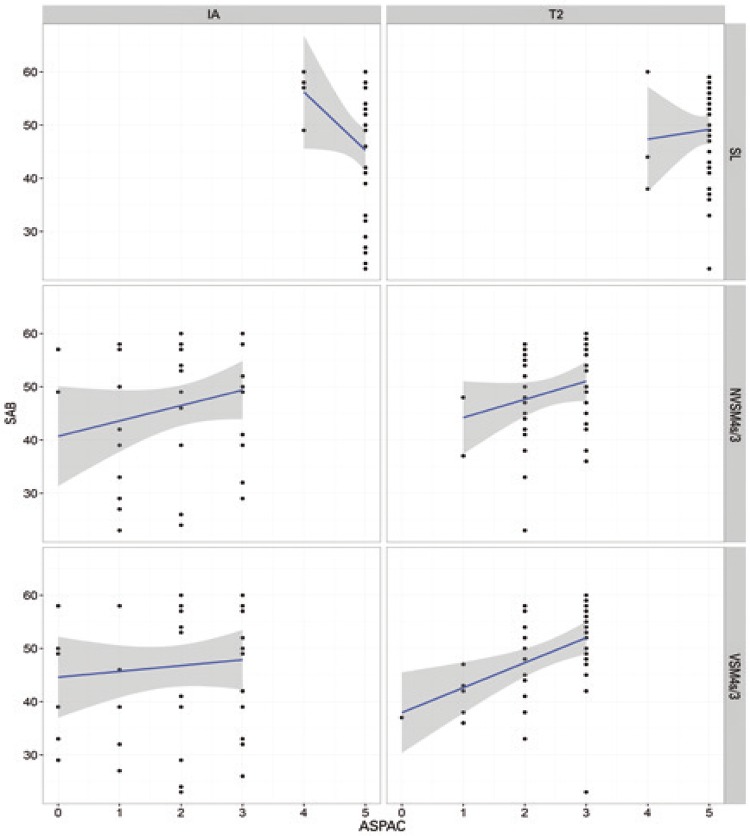
Relation between Scale of Auditory Behaviors scores and performance in *Avaliação Simplificada do Processamento Auditivo Central* in the Multisensory Group at initial (IA; left column) and final (T2; right column) time points

SL: sound localization; NVSM4s/3: nonverbal sequence memory test - 4 sounds/3 sequences; VSM4s/3: verbal sequence memory test - 4 sounds/3 sequences; SAB: Scale of Auditory Behaviors; ASPAC: *Avaliação Simplificada do Processamento Auditivo Central*.

**Figure 4:**
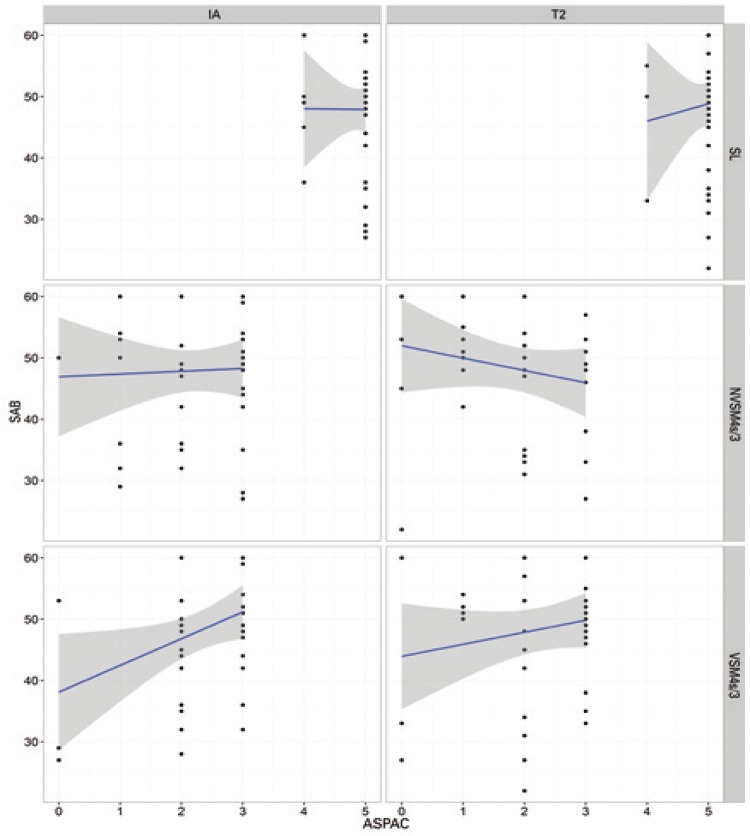
Relation between Scale of Auditory Behaviors scores and performance in *Avaliação Simplificada do Processamento Auditivo Central* in the Control Group at initial (IA; left column) and final (T2; right column) time points

SL: sound localization; NVSM4s/3: nonverbal sequence memory test - 4 sounds/3 sequences; VSM4s/3: verbal sequence memory test - 4 sounds/3 sequences; SAB: Scale of Auditory Behaviors; ASPAC: *Avaliação Simplificada do Processamento Auditivo Central*.

ANOVA of IA data failed to reveal differences between groups. Therefore, potential biases (*i.e*., groups with comparatively good or poor pre-training performance in auditory processing tasks) were excluded. Interactions (p<0.05) between SAB scores and ASPAC performance in different groups and at different time points (IA and T2) are shown in table 4 and figures 1 to 4.

**Table 4:** Interaction among the variables of Scale of Auditory Behaviors and *Avaliação Simplificada do Processamento Auditivo Central* per groups studied and initial (IA) and final (T2) time points

Groups	Tests	IA				T2			
	
		Correlation	SL	VSM4s/3	NVSM4s/3	Correlation	SL	VSM4s/3	NVSM4s/3
**MAG**	SAB	ρ (rho)	-0.092	0.239	0.192	ρ (rhô)	-0.080	0.236	0.000
		p	0.285	0.066	0.115	p	0.309	0.069	0.499
		n	41	41	41	n	41	41	41
**AMG**	SAB	ρ (rho)	0.083	0.068	0.261	ρ (rho)	0.204	0.022	0.330
		p	0.306	0.338	0.052	p	0.104	0.446	0.019*
		n	40	40	40	N	40	40	40
**MSG**	SAB	ρ (rho)	-0.266	0.103	0.299	ρ (rho)	0.044	0.465	0.224
		p	0.046*	0.262	0.029*	p	0.393	0.001*	0.079
		n	41	41	41	N	41	41	41
**CG**	SAB	ρ (rho)	0.013	0.239	-0.008	ρ (rho)	0.079	0.187	-0.234
		p	0.468	0.069	0.479	p	0.314	0.127	0.076
		n	40	40	40	n	40	39	39

Spearman correlation. ρ (rho) is the p value correlation. * Statistically significant.

LSL: sound localization; VSM4s/3: verbal sequence memory test - 4 sounds/3 sequences; NVSM4s/3: nonverbal sequence memory test - 4 sounds/3 sequences; MAG: Motor/Auditory Group; AMG: Auditory/Motor Group; MSG: Multisensory Group; CG: Control Group; SAB: Scale of Auditory Behaviors; IA: pre-training; T2:post-training.

No interactions between performance in ASPAC and SAB scores were noted in the MAG or CG at any of the time points. In contrast, interactions were detected in the AMG (MSnV4s test) at both time points, *i.e*., SAB scores increased as performance in ASPAC improved. Interactions between variables were also detected in the MSG (SL test, negative correlation at IA; MSV test, positive correlation at T2). Pre- and post-training (IA and T2) differences in experimental groups were not detected in the CG.

## DISCUSSION

Experimental groups in this study achieved higher SAB scores following training, with significant pre- and post-training differences in the MAG. Therefore, auditory and motor training seems to have promoted positive auditory behavior changes, as rated by school teachers.^(^ ^13^ ^,^ ^23^ ^,^ ^24^ ^)^

Mean MAG pre-training scores are suggestive of CAPD.^(^ ^13^ ^)^ However, improved auditory behavior in treated groups following 8 hours of stimulation points to training effectiveness and emphasizes the significance of tools aimed at assisting school teachers with recognition of behaviors associated with learning dysfunction, *i.e*., observational tools beyond traditional pedagogical assessment methods. According to previous studies, school teachers are often ill-informed about reading and writing disorders and related causes, possibly due to lack of specific higher education training.^(^ ^26^ ^,^ ^27^ ^)^

All groups performed well in ASPAC SL test at IA. Low variability in test responses may have reflected the fact that mean IA scores were within normal ranges. Lack of changes in sound localization skills in school children with reading and writing disabilities has been reported.^(^ ^12^ ^)^

Changes detected in MSV and MSnV-4 sound tests at IA in this study have been described in school children with learning difficulties, with and without CAPD.^(^ ^12^ ^,^ ^14^ ^)^ Therefore, lack of temporal ordering skills is vital for language processing. Children in the experimental groups in this trial showed normal temporal ordering skills at T2, with significant differences between IA and T2.

At the age of three years, children are already capable of repeating three-syllable sequences, and can handle longer sequences at seven years, with improved performance as they grow. At the age of six years, children are capable of memorizing four musical (nonverbal) sounds played in a given sequence, with successful performance in at least two out of three attempts.^(^ ^7^ ^)^

Intergroup IA comparisons revealed similar performance in auditory processing tasks and groups submitted to training performed significantly better in the MSnV test compared to the CG. Improved temporal ordinance performance in school children submitted to auditory stimulation has been reported.^(^ ^20^ ^)^

Initial changes in temporal ordinance tests in this study may be a predictor of academic difficulties. Improved skills after stimulation T2 of experimental groups demonstrate positive effects of the therapeutic program proposed, with relevant contribution to better neurobiological learning in school settings.

Hence, according to the neuroplasticity concept, stimulation and experience led to activation and reinforcement of specific neural pathway, thereby supporting children in the recognition of novel patters and acquisition of new context and skills.^(^ ^15^ ^-^ ^18^ ^,^ ^28^ ^)^ This finding is consistent with the idea of motor and sensory system improvement in response to experience and learning. These systems connect recognition of environmental stimuli with a wide range of motor responses. It can therefore be argued that reading and writing disabilities are not limited to the verbal domain.^(^ ^28^ ^)^

In this study, auditory and motor stimulation in school settings led to rapid changes in auditory behavior. *Avaliação Simplificada do Processamento Auditivo Central* was thought to be a sensible tool for detection of such behavioral variations and may be effectively applied to screen for CAPD in school children.^(^ ^7^ ^,^ ^13^ ^,^ ^19^ ^,^ ^21^ ^)^

*Avaliação Simplificada do Processamento Auditivo Central* variables were significantly correlated with SAB questionnaire responses given by school teachers in the AMG (MSnV test) and MSG (MSnV and SL tests), both at IA and T2. As regards other significantly correlated skills at IA, school teachers failed to notice effects of stimulation.

Correlations between experimental group SAB scores and ASPAC performance revealed small but positive variations in IA and T2 means, suggesting beneficial effects of training. The lack of positive variations in the CG should be emphasized.

Relations between child behavior as perceived by school teachers and corrauditory test responses in this study are consistent with findings of Nunes et al., - *i.e*., the better results auditory processing tests, the higher the SAB scores.^(^ ^13^ ^)^

Findings from this study suggest the therapeutic program proposed^(^ ^25^ ^)^ and implemented in school settings can be used to characterize and dinstinguish school children with actual CAPD evidence, who need specialized therapy.^(^ ^13^ ^)^ Also, aplication of behavioral tests in scholl settings may help teachers recognize signs of auditory changes in children.^(^ ^7^ ^,^ ^22^ ^-^ ^24^ ^)^ Self-assessment questionnaires and the Portuguese version of the SAB have been employed in recent studies; still, validation for the Brazilian population is lacking and may be regarded as a limitation of this study.

Multisensory experiences contribute to the development of perception mechanisms, with widely demonstrated impacts on cognition. Multisensory integration between motor and sensory systems reach maturity around the age of 11 years;^(^ ^16^ ^,^ ^28^ ^)^ hence the relevance of stimulation programs focusing on primary education and amenable to implementation in school settings as a means to support children with reading and writing difficulties. Interdisciplinary approaches for greater inclusion of children with learning disabilities should be encouraged.

The sample in this study was selected at random. However, stimulation programs aimed at children with special educational needs, including longer sessions and teacher capacitation, may contribute to rehabilitation and development of the reading and writing skills.

## CONCLUSION

School children in this study performed similarly when first submitted to the *Avaliação Simplificada do Processamento Auditivo Central*. Auditory and motor training led to significant auditory and motor skill improvements, which were also perceived by school teachers, as shown by Scale of Auditory Behaviors scores. The intervention model described proved to be an effective tool amenable to application in school settings.

## Appendix

**Appendix 1:** . Intervention models applied to experimental groups, per task and skill

Group	Week	Session	Task	Auditory stahe	Motor stage
MSG	1	1 and 2	Identification of instrument sounds with movement towards the sound and saying instrument name (blindfolded) Relate instrument sound to position in space (front, back, right and left) and move in the direction proposed (blindfolded) Expose children to environment sounds (*e.g*., door close and crumbling paper sounds). Blindfolded, name sound, indicate sound source and take a step towards it Divide vertical plane: RA, RB/LA and LB. Throw a tennis ball in the direction and sequence requested (*e.g*., RA, LB, DB, LA etc.)	Sound discrimination and localization	Spatial orientation
MSG	2	3 and 4	Zigzag Hula hoop trajectory. Touch left and right hoops with left and right foot respectively, according to metronome beat pattern Follow the same trajectory; place right foot in right hoop and left foot in left hoop when hearing rattle and bell sound, respectively Same hoop trajectory; match fruit name with jump to the right and number with jumps to the left Play blind man’s buff following voice cues to the right, front, back and left	Sound discrimination and localization	Dynamic balance
MSG	3	5 and 6	Child on a stool. Musical instrument sound sequence; throw ball upwards and name sounds heard (use four musical instruments) Repeat activity with semantic category (*e.g*., fruits); use the same four stimuli in alternate sequence Stand on a masking tape line with one foot in front of the other and blindfolded; name nonverbal sound sequences Stand on a line with one foot in front of the other and holding a ball. Alternate verbal and nonverbal sounds in the same sequence: verbal sounds, kick ball on the right; nonverbal sounds, kick ball on the left while repeating sequence	Temporal ordinance	Static balance and visuomotor coordination
MSG	4	7 and 8	Hands on the floor, feet on the wall; sustain position while mediator presents sound sequence; get down from the wall and name sounds heard. Begin with three nonverbal stimuli and increase With the body suspended, repeat semantic category in sequence while moving to the right or left. Begin with three verbal stimuli. Draw a line (1.5m) on the floor. Number sequence. Child must walk to the end of the line, forward and backward, while repeating number sequence heard. Begin with three stimuli and increase. Sitting in circle, each child adds an item to a supermarket list in sequence – repeat item sequence and name the child who added the item	Temporal ordinance	Tonus persistence
MSG	5	9 and 10	Music playing on the radio; sequence of verbal commands asking attention and naming of certain body parts that should contact the ground in the lying position. When relaxing, child must name points of contact in the same order dictated by therapist Prone position. Focus on mediator, who will ask for change of head position from right to left every 10 seconds, while ignoring a history played in the background at moderate intensity Instrumental background music, blindfolded child standing still on a line, one foot in front of the other. Instruments played in different directions. Ignore noise and indicate source of sound Background music child blindfolded and standing still. Body part sequence dictated by mediator should be touched in reverse order. Begin with three stimuli	Figure-ground, temporal ordinance and sound localization	Segmental limb awareness
MSG	6	11 and 12	Supine position, arms along the body. Word sequence (semantic class and background noise). Ignore noise, repeat sequence in the same order with arms raised, then relax arms. Repeat with legs and arms using number sequence. Sequence dictated by mediator in silent conditions and repeated by child in noisy conditions Two parallel lines (4m long each). Familiar songs and background noise. Hold a tennis ball between body parts. Walk between lines while holding ball. Stop and complete background song In circle, with a ball. Child sings a nursery rhyme and, at mediator’s command, throws the ball to a friend, who then resumes singing	Temporal ordinance and auditory closure	Contraction and relaxation
MSG	7	13 and 14	Play two sound stimuli on a keyboard with different time intervals. Child must kick the ball according to sounds heard (one or two) and verbalize Same strategy; child must kick ball with right or left hand when hearing one and two sounds, respectively Paired sounds – long/upward, short/downward. Child must throw the ball up into the air or down accordingly Tennis ball and wall divided into right and left halves. Short and long sound sequence. Short sound, kick ball on right side; long sound, kick ball on left side	Auditory discrimination and temporal ordinance	Visual and motor coordination
MSG	8	15 and 16	Move around associating step count and distance with number of words contained in sentences presented by mediator Two parallel lines (3m long each). Walk between lines with step count determined according to number of syllables in selected words (*e.g*., chocolate, agent and success) Walk around the room with a metronome, count 4 beats and stop (stand) for 4 beats; vary speed according to metronome beat pattern Words required to complete sentences in a story written on paper and scattered on the floor. At each pause (recorded story), child must find the word that fits	Auditory detection and closure	Space-time perception
AMG/MAG	1/5	1 and 2/9 and 10	Expose child to different environment sounds (*e.g*., door close, chair dragging etc.); name sound heard (blindfolded) Identify one of the sounds heard (agogo, *coco*, rattle, bell) by pointing to sound source and naming instrument (blindfolded) Play instruments in horizontal plane (right/left/front/back); indicate source of sound (blindfolded) Play blind man’s buff according to voice cues directing players to the right, left, front or back	Sound detection, discrimination and localization	-
AMG/MAG	2/6	3 and 4/11 and 12	Expose child to different environmnet sounds (*e.g*., crumbling paper, key, ball kicking). Reproduce sounds heard (in different order) and increase sequence progressively. Write down the number of stimuli the child is able to reproduce Sitting in circle, each child adds an item to a supermarket list in sequence repeat item sequence and name the child who added the item Repeat nonverbal sound (musical) sequence and name instruments in the sequence they were played Movable alphabet letters and semantic class labels. The child picks a letter and a class, then says one word beginning with that letter within that semantic class	Temporal ordinance	-
AMG/MAG	3/7	5 and 6/13 and 14	Instruments played in different directions with background noise (*e.g*., out of tune radio). Ignore noise and point to the direction of sound source Four-word (fruits) sequence dictated with radio playing at moderate intensity. Repeat sequence adding one word to sequence (up to ten words per sequence) Four-number sequences dictated in silent conditions; child repeats in noisy conditions. Change numbers in sequence *Play familiar songs with rhymes (e.g*., nursery rhymes) in full. In circle, passing a ball; when music stops the one holding the ball must complete song	Figure-ground, auditory closure and temporal ordinance	-
AMG/MAG	4/8	7 and 8/15 and 16	Play two sound stimuli on a keyboard with different time intervals. Say whether one or two sounds were heard. Gradually reduce interval between sounds Use consonant cluster words (*e.g*., plate, flute, plan, white, light etc.). Present words in two manners: correct manner, then adding one vowel before consonant (*e.g*., plate and palate); indicate sound heard saying whether the correct form was the first or second Sentence read by mediator. Child must indicate the number of words in each sentence. Gradually increase In circle. Mediator reads a story; when mediator stops selected child must complete sentence to make sense	Temporal ordinance and phonological awareness	-
AMG/ AMG	5/1	9^h^ and 10/ 1 and 2	Identify different positions in space, front, back, right, left, inside, outside and around a circle drawn with chalk. Reference: to locate teacher’s position in space – only verbal command with movement variation Identify and take one step to different positions in space while facing mediator: mediator gives verbal command and moves at the same time Two parallel masking tape lines; jump inside, outside, on top, to the right and to the left according to mediator’s command, who shall change position in space Divide vertical plane: RA, RB/LA and LB. Throw tennis ball as requested (*e.g*., RA, LB, DB, LA etc.)	-	Spatial orientation and dynamic balance
AMG/MAG	6/2	11 and 12/3 and 4	Sit quietly on a chair while manipulating ball (throw ball up into the air and catch it) On the floor, (throw ball up into the air while moving to the right, left, front and back as requested by mediator Masking tape line on the floor (1.5m). Walk on line placing one foot in front of the other while kicking ball to the right or left as opposite front foot hits the ground Feet on the wall, hands on the floor. Sustain position for 10 seconds then relax. Increase time by 10 seconds per round up to five rounds. Unsuccessful round may be repeated once	-	Static balance, visuomotor coordination and tonus persistence
AMG/MAG	7/3	13 and 14/5 and 6	In pairs. Between two masking tape lines, walk while holding the ball between body parts (*e.g*., head, shoulder, back, knee, foot, elbow, hip, wrist, ear and thumb) Standing on a line, one foot in front of the other, touching it. Blindfolded. Stand still for 30 seconds. At each successful attempt, increase standing time by 10 seconds Standing on a line, one foot in front of the other, kick ball and catch it alternating hands In pairs, throw a ball towards a pin to knock it down. Write down the number of hits out of ten attempts and change position	-	Segmental limb awareness; contraction and relaxation
AMG/MAG	8/4	13 and 14/5 and 6	Using a tennis ball, child must throw ball with right and left hand in sequence, touching the floor, ceiling, right and left walls and not letting the ball drop on the floor One ball in each hand; throw ball up into the air and down with the right or left hand (*e.g*., right hand throws ball up, left hand throws ball down) Two parallel lines (5m long each; walk from one side to the other while counting steps required. Cover the same distance with varying step count, as requested by mediator. Shorten or lengthen stride Walk between lines with metronome; walk from one side to the other at metronome beat pattern. Once completed, repeat with faster beat pattern	-	Visuomotor coordination and space-time perception

MSG: Multisensory Group; RA: right above; RB: right below; LA: left above; LB: left below; AMG: Auditory/Motor Group; MAG: Motor/Auditory Group.
